# A phase 1 trial of human telomerase reverse transcriptase (hTERT) vaccination combined with therapeutic strategies to control immune-suppressor mechanisms

**DOI:** 10.3389/ebm.2024.10021

**Published:** 2024-01-31

**Authors:** Nahid Zareian, Oleg Eremin, Hardev Pandha, Richard Baird, Vineet Kwatra, Gabriel Funingana, Chandan Verma, Desmond Choy, Steven Hargreaves, Pejvak Moghimi, Adrian Shepherd, Dileep N. Lobo, Jennifer Eremin, Farzin Farzaneh, Shahram Kordasti, James Spicer

**Affiliations:** ^1^ School of Cancer and Pharmaceutical Sciences, King’s College London, London, United Kingdom; ^2^ Nottingham Digestive Diseases Centre, NIHR Nottingham Biomedical Research Centre, Queen’s Medical Centre, Nottingham University Hospitals NHS Trust and University of Nottingham, Nottingham, United Kingdom; ^3^ Department of Microbiology and Cellular Sciences, University of Surrey, Guildford, United Kingdom; ^4^ Cancer Research UK Cambridge Centre, Cambridge, United Kingdom; ^5^ Research Department of Pathology, UCL Cancer Institute, Faculty of Medical Sciences, University College London (UCL), London, United Kingdom; ^6^ The Institute of Structural and Molecular Biology (ISMB), Birkbeck, University of London, London, United Kingdom; ^7^ MRC Versus Arthritis Centre for Musculoskeletal Ageing Research, Queen’s Medical Centre, School of Life Sciences, University of Nottingham, Nottingham, United Kingdom

**Keywords:** hTERT, vaccination, TLR agonist, phase-1 trial, CD8^+^ T-cells

## Abstract

The presence of inhibitory immune cells and difficulty in generating activated effector T cells remain obstacles to development of effective cancer vaccines. We designed a vaccine regimen combining human telomerase reverse transcriptase (hTERT) peptides with concomitant therapies targeting regulatory T cells (Tregs) and cyclooxygenase-2 (COX2)-mediated immunosuppression. This Phase 1 trial combined an hTERT-derived 7-peptide library, selected to ensure presentation by both HLA class-I and class-II in 90% of patients, with oral low-dose cyclophosphamide (to modulate Tregs) and the COX2 inhibitor celecoxib. Adjuvants were Montanide and topical TLR-7 agonist, to optimise antigen presentation. The primary objective was determination of the safety and tolerability of this combination therapy, with anti-cancer activity, immune response and detection of antigen-specific T cells as additional endpoints. Twenty-nine patients with advanced solid tumours were treated. All were multiply-pretreated, and the majority had either colorectal or prostate cancer. The most common adverse events were injection-site reactions, fatigue and nausea. Median progression-free survival was 9 weeks, with no complete or partial responses, but 24% remained progression-free for ≥6 months. Immunophenotyping showed post-vaccination expansion of CD4^+^ and CD8^+^ T cells with effector phenotypes. The *in vitro* re-challenge of T cells with hTERT peptides, TCR sequencing, and TCR similarity index analysis demonstrated the expansion following vaccination of oligoclonal T cells with specificity for hTERT. However, a population of exhausted PD-1^+^ cytotoxic T cells was also expanded in vaccinated patients. This vaccine combination regimen was safe and associated with antigen-specific immunological responses. Clinical activity could be improved in future by combination with anti-PD1 checkpoint inhibition to address the emergence of an exhausted T cell population.

## Impact statement

Difficulty in generating activated effector T cells and the presence of inhibitory immune cells are obstacles to development of tumour vaccines. hTERT is overexpressed in >90% of malignancies. We conducted a Phase 1 trial, in which patients with different multiply-pretreated solid tumours were vaccinated with an hTERT-derived 7-peptide library, with a novel adjuvant strategy, selected to ensure presentation by both HLA class-I and class-II in 90% of a European population. Adjuvants were Montanide and topical TLR-7 agonist, to optimise antigen presentation. Oral low-dose cyclophosphamide, to modulate regulatory T cells, was combined with celecoxib to block cyclooxygenase-2-mediated immunosuppression. The primary objective was determination of vaccine/adjuvant safety and tolerability, with immune response and detection of antigen-specific T cells as exploratory endpoints. This vaccine was safe. The data demonstrates the induction of immunological responses, including clonal expansion of hTERT reactive T cells and clinical disease stabilisation for over 6 months in a quarter of these therapy-resistant patients.

## Introduction

Immune therapy of cancer using vaccination strategies has had limited success to date, due to low immunogenicity of selected antigens, poor stimulation of effector T cells, and incomplete tumour specificity. Human telomerase reverse transcriptase (hTERT) is expressed at elevated levels in >90% of human tumours [1–3], and offers a potential target for active vaccination mediated immune therapy of cancer. Recognition of hTERT peptides by αβ T cells is HLA-restricted, requiring the selection of subsets of patients with specific HLA haplotypes (e.g., HLA-A2) in previous hTERT vaccination studies. In order to maximise eligibility for vaccination, we chose to use an hTERT peptide library compatible with multiple HLA haplotypes. This library was predicted to be suitable for direct presentation by at least one HLA class-I and one HLA class-II allele in over 90% of a European population [4–6]. The uptake and processing of long peptides, which involves the active uptake, processing and presentation of such peptides by the professional antigen presenting cells, require no HLA-selection [7]. No long peptides were included in our hTERT library.

The selected hTERT library consisted of seven peptides identified as immunogenic self-antigens that are predicted to bind directly, without intracellular processing, to common HLA proteins (4 to class I and 3 to class II) [8–12]. These were subsequently confirmed to have a high binding affinity to the predicted HLAs.^1^ We also used a novel adjuvant strategy. Adjuvants employed were Montanide (ISA-51 VG), a water-in-oil emulsion composed of a mineral oil and a mannide mono-oleate surfactant, injected intradermally with the peptide library. In addition, imiquimod, an agonist for toll-like receptor 7 (TLR-7) was applied topically, in order to stimulate innate and acquired immune responses [13]. Each 3-weekly dose of vaccine was preceded by oral low dose cyclophosphamide (50 mg twice daily for the first 10 days of each cycle), in order to reduce immune suppressive regulatory T cells (Tregs) and myeloid derived suppressor cells (MDSCs) [14–17]. A cohort of 15 patients also received celecoxib, to inhibit cyclooxygenase-2 (COX-2), with the intention of suppressing pro-tumourigenic prostaglandin levels. This trial is the first step in a strategy termed “Combined Adjuvants for Synergistic Activation of Cellular immunity” (CASAC) [18, 19].

The aim of this study was to investigate the safety of this vaccine, and also to assess whether it could induce an antigen-specific cell-mediated immune response in a cohort of patients with advanced, therapy resistant solid tumours. Multi-dimensional immunophenotyping and T cell receptor (TCR) sequencing analysis were used to assess the specificity and magnitude of post-vaccination immunological responses.

## Materials and methods

### Trial design

We conducted a Phase 1 clinical trial in patients with advanced metastatic solid tumours for whom further standard therapy was unavailable or not suitable. This was an open label, fixed dose trial (see trial flow chart, Supplementary Figure S1). The primary objective of the study was to assess the safety and tolerability of the vaccine and associated therapy. Secondary objectives were to document vaccination-mediated stimulation of antigen-specific cellular immune responses (CD4+/CD8+ T cell), and to assess any clinical evidence of anti-tumour activity. The trial was approved by the Medicines and Healthcare products Regulatory Agency (EudraCT Number 2014-003025-18). Patients were recruited and treated at three UK cancer centres.

### Patients

Eligible patients were aged ≥16, had histologically proven solid cancer, prior anti-cancer treatment completed ≥4 weeks previously, with no further suitable anti-cancer therapy option being available, and with a WHO performance status ≤2. Excluded comorbidities were autoimmune disorders, ongoing immune-suppressive therapy including steroids, central nervous system malignancies (primary and secondary), coronary artery disease, other major cardiac disease (documented left ventricular ejection fraction <50%), poorly controlled hypertension (diastolic >100 mmHg), and requirement for anticoagulation.

### Trial treatment

Patients received 10 days of oral cyclophosphamide (50 mg twice daily orally on days 1–10), followed by the hTERT peptide vaccination emulsified in the Montanide adjuvant delivered intradermally on day 15 of 3-weekly cycles, and topical imiquimod (Meda Pharmaceuticals, Stockholm, Sweden). Of the 29 patients recruited, 15 were allocated non-randomly to receive additional continuous daily oral celecoxib, provided there was no contra-indication to receiving non-steroidal anti-inflammatory drugs. Seven GMP grade peptides were synthesised (4 class-I hTERT peptides: designated p324/325, p611, p865, p973; and 3 class-II hTERT peptides: p672, p766, p1123; designation numbers signify amino acid residue in the full-length hTERT protein; American Peptide Company, Sunnyvale, CA, United States) and transferred to a GMP facility (Rayne Institute, King’s College London), where peptides were formulated as a mixture at equimolar concentrations (10 μg/mL). The hTERT peptide sequences, frequency of HLA linkage (haplotype population prevalence), and their avidity scores (SYFPEITHI database for MHC-peptide-prediction, University of Tubingen, Germany^1^) are listed in Supplementary Figure S2. These hTERT peptides bind with high affinity to specific HLA molecules; the vaccine was designed to permit the presentation of at least one Class-I and one Class-II peptide by the HLAs present in >90% of patients.

Immediately prior to vaccination, the seven peptides in 1 mL phosphate buffered saline were thawed and emulsified at the bedside by mixing with 1 mL Montanide ISA-51 VG. The emulsion was injected intradermally (2 mL) at multiple sites, at each injection site with a volume of 0.1–0.2 mL, in a 5 cm × 5 cm area of the anterior abdominal wall. New vaccination sites were used for each subsequent cycle. Patients received up to 8 cycles of vaccination in the absence of tumour progression or intolerable toxicity. Patients considered to be benefiting from treatment were allowed to continue beyond eight cycles.

### Patient evaluation

Patients were clinically assessed at baseline and every cycle for disease symptoms and treatment-related adverse events. This included analysis of blood samples for renal and liver function tests, full blood count and, where relevant, tumour markers. Serial CT scans performed every 3 cycles were used to evaluate measurable disease. A sub-set of patients underwent additional intradermal injections (100 μL) of peptide mixture only (without either Montanide adjuvant or imiquimod) in cycles 1, 3 and 6 to investigate delayed type hypersensitivity (DTH) responses. Peripheral blood samples were collected from patients prior to, during and post-vaccination, to characterise patients’ cellular immune response to vaccination.

### Immunophenotyping

Frozen peripheral blood mononuclear cells (PBMCs) were used for immunophenotyping [Supplementary Material (materials and methods)]. To assess the specificity of the response to vaccine peptides, cells obtained from HLA-A0201 patients were challenged *in vitro* with the hTERT peptide library overnight, in the absence of any adjuvants but in the presence of protein transport inhibitor GolgiPlug (BD Biosciences, Erembodegem, Belgium). An irrelevant WT1-derived HLA-A0201-presented peptide 126–134 (RMFPNAPYL, referred to as RMF), served as a control. Cells were subsequently fixed, permeabilised, and stained with a selection of antibodies specific to T cells, regulatory T cells, T cell degranulation, T cell activation, immune check-points, T cell apoptosis markers and cytokines: CD3, CD8, CD4, CD45RO, CD127, CD25, CD107a, CD137, CD69, CD154, CD95, TCR Vd2g9, FoxP3, HLA-DR, CTLA-4, PD-1, TIM-3, IL-2, TNF-α and IFN-γ.

### *Ex vivo* T cell stimulation assay

Patients’ PBMCs were subjected to two weekly cycles of *ex vivo* stimulation in culture, in the presence of hTERT peptides at 70 μg/mL in X-Vivo 15 (Lonza, Bioscience) to allow more detailed immunopenotyping of antigen-specific lymphocyte populations. These cultures were supplemented with 5 ng/mL of interleukins (IL)-4 and -7 on the first and second day. The culture was maintained for 2 weeks with addition of fresh medium, containing 40 IU/mL IL-2 (Peprotech, London, United Kingdom), every second day. After 2 weeks, genomic DNA was extracted for TCR-β chain analysis by high-throughput sequencing.

### TCR-β chain sequencing

DNA was extracted (Qiagen’s DNeasy mini-columns, QIAGEN Inc., Germantown, MD, United States) from PBMC either without *in vitro* stimulation or after two rounds of hTERT peptide stimulation over 2 weeks. All TCR-β characterisation was performed by Adaptive Biotechnologies Corp (Seattle, WA, United States), using the ImmunoSEQ TCR-β human assay [Supplementary Material (materials and methods)].

### Statistical analysis

The analyses of statistical significance of the differences between groups (pre- and post-vaccination) were performed using the Brown-Forsythe test. All other tests, unless otherwise indicated, were performed using the Student’s t-test. A *p*-value ≤ 0.05 was considered statistically significant. For the statistical analysis of TCR-β chain similarity index, all Hamming distances were calculated for each of the pairwise patient-combinations [20]. The average and the standard deviation values for Hamming distances were calculated to generate standard error.

## Results

### Safety and anti-tumour activity

Twenty-nine patients with different solid tumour types underwent vaccination (Table 1). All patients had received prior systemic therapy, in many cases multiple lines of treatment; no further standard therapy was available or considered suitable. The study treatment was generally very well tolerated and almost all treatment-related adverse events were low grade (≤2). The most common events were injection site reactions, fatigue and nausea (Table 2). Erythema and limited cutaneous induration confined to the vaccinated area was commonly seen, usually maximal after 1 week and gradually fading thereafter. Two patients experienced a hypersensitivity reaction at the vaccination site with a much more marked area of localised oedema and overlying erythema, associated with pruritis. These patients were withdrawn from the study. No other treatment discontinuations due to treatment-related toxicity occurred. In the subset of patients who received intradermal injections of peptide only (without the Montanide adjuvant) no DTH responses were observed, so the injection site reactions were likely to be due to the use of adjuvant. There were no RECIST radiological or tumour marker responses. Seven patients (24%) had disease that remained stable for at least 6 months after trial initiation.

**TABLE 1:** Summary of patient characteristics.

Tumour (*n* = 29)	
Colorectal	12
Prostate	5
Lung	2
Pancreas	2
Breast	2
Oesophago-gastric	2
Cervix	1
Mesothelioma	1
Ovary	1
Renal	1
Age (median, rage)	62 (35–74)
Male	66%
Female	34%

Characteristics of twenty-nine patients with different solid tumour types that underwent vaccination.

**TABLE 2:** Treatment-related adverse events.

*n* (%)	Grade 1/2	Grade 3
*Injection site reaction	14 (45%)	
Fatigue	7 (23%)	
Nausea	5 (16%)	
Diarrhoea	3 (10%)	
Lymphocytopenia	3 (10%)	
ALP elevation	1 (3%)	1 (3%)
Anaemia	2 (6%)	
Anorexia	2 (6%)	
Weight loss	2 (6%)	

Adverse events reported as related to study medications in more than one patient.

No grade 4 events were observed.

ALP, alkaline phosphatase.

*Injection site reaction includes erythema, oedema, pruritis and discharge.

### Post-vaccination immune phenotyping of patient PBMCs (immune response to vaccine)

We first used screening with conventional flow cytometry, using a limited number of antibodies (CD3, CD8, CD107a, CD137, TNF-α, IFN-γ, and IL-2) to identify potential T cell responses following vaccination. Sufficient serial PBMC samples were obtained from 24 patients enrolled in the trial for immunophenotyping. We characterised changes in immune cell populations of un-challenged PBMCs from patients following vaccination. The proportions of both memory CD4^+^ and CD8^+^ T cells (CD45RO^+^) were significantly higher in the post-vaccination samples (*p* = 0.01 and *p* = 0.04, respectively; data not shown).

We then re-challenged cells *ex vivo* with hTERT, or an irrelevant peptide as control. The proportions of both CD4^+^ and CD8^+^ cells expressing cytokines IL-2 (*p* = 0.01, *p* = 0.02), TNF-α (*p* = 0.03/*p* = 0.02) and IFN-γ (*p* = 0.03/*p* = 0.02) increased significantly compared to pre-vaccination levels in response to re-challenge with hTERT, but not in response to the irrelevant peptide.

### Deep immunophenotyping analysis of unchallenged/challenged patient samples

To further analyse the phenotype of T cells following vaccination, with and without hTERT rechallenge, we used a multi-colour flowcytometer with the capacity to analyse 20 markers [using multidimensional flow cytometry with a panel of 17 markers (CD3, CD8, CD4, CD45RO, CD127, CD25, CD107a, CD137, CD69, CD154, CD95, TCR Vd2g9, FoxP3, HLA-DR, CTLA-4, PD-1, TIM-3, TNF-α, IFN-γ and IL-2)]. (BD FACSymphony™ flowcytometer), as well as an in-house automated clustering algorithm for analysis of multidimensional flowcytometry data^2^ as described in Supplementary Material (Materials and Methods). Initially, various clusters of CD8^+^ and CD4^+^ T cells were identified in unchallenged samples by visualization of t-distributed stochastic neighbour embedding (viSNE).

**FIGURE 1:**
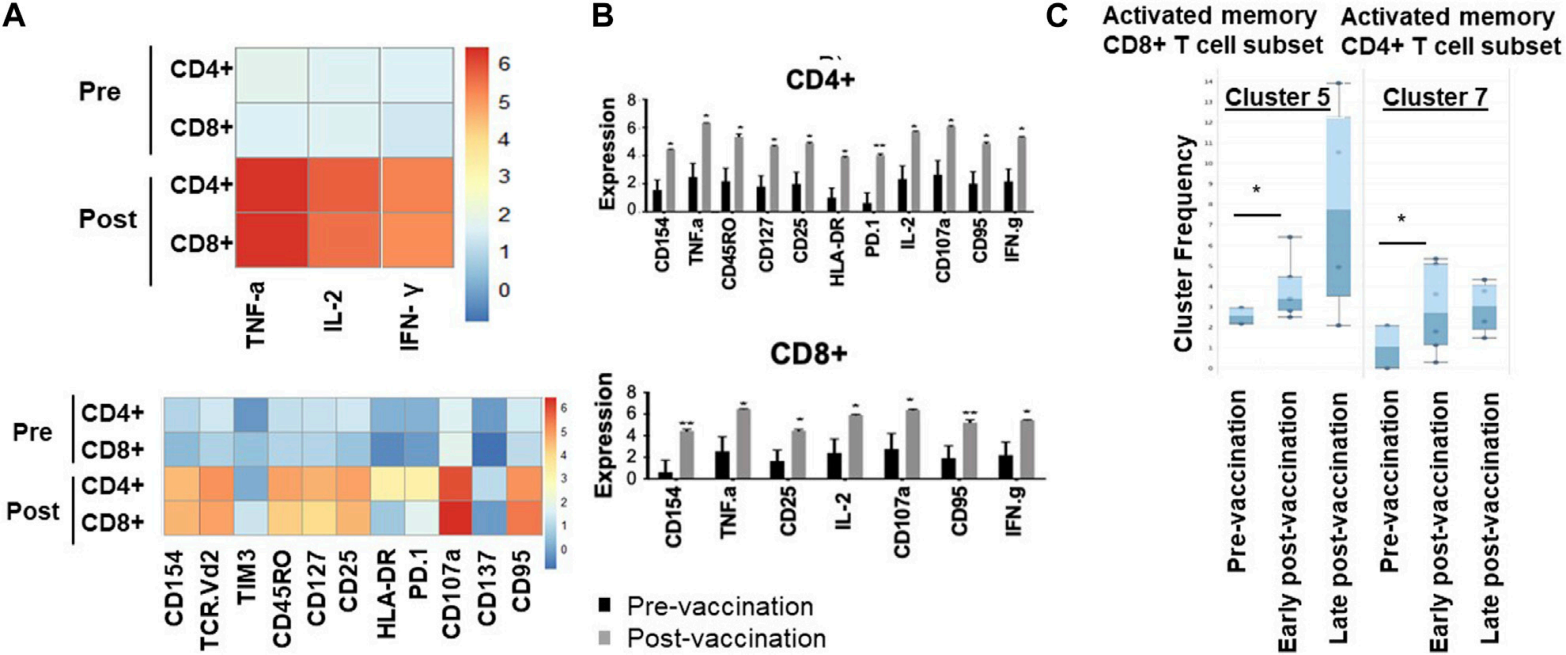
Memory and activated hTERT-responsive CD4^+^ and CD8^+^ T cells increase post-vaccination. **(A)** Median expression of the selected markers in pre- and post-vaccination PBMCs from 24 patients as identified by clustering algorithm CytoClustR on a subset of 400,000 cells proportionally selected from all samples. Each row represents a cell cluster and each column represents a marker. Heatmap plot generated from SPADE analysis. Expression values were transformed using the arcsin function in a cofactor of 5. The plot shows a difference in the immunological signature between pre- and post-vaccination T lymphocytes, a phenotype that shows increased presence of memory (CD45RO) activated (CD107a, HLA.DR) CD8^+^ T cells. These stimulated cells appear to express inhibitory co-receptors (PD-1, TIM3), as well as CD95 (APO-1/Fas), which are known to be increased in post-vaccination T cells. **(B,C)** Quantitation of T cell activation markers in different clusters of unstimulated cells, with significantly increasedexpression post-vaccination in a cluster (cluster 5) CD8^hi^, HLADR^hi^, PD-1^hi^, CTLA4^hi^, TIM-3^hi^ CD45RO^hi^, CD95^hi^, CD107a^hi^, CD137^hi^. Error bars are standard error of mean. Two-way Anova analysis of variance test was used for statistical analysis. *p < 0.05.

We have then used downstream clustering methods to determine the detailed phenotypes of the expanded T cells post-vaccination in both the unchallenged state and following *in vitro* re-challenge of the PBMC, respectively. In the unchallenged PBMC, expanded CD4^+^ T cells expressed significantly higher levels of activation markers after vaccination, including CD154 (*p* = 0.01), HLA-DR (*p* = 0.006), CD107a (*p* = 0.005) as well as the exhaustion marker PD1 (*p* = 0.01), and Fas marker CD95 (*p* = 0.007); exhausted CD4^+^ T cells display reduced production of effector cytokines such as TNF-α and IFN-γ and increased Fas death markers (Figure 1).

Similarly, post-vaccination CD8^+^ T cells expressed significantly higher levels of activation markers including CD154 (*p* = 0.02) and CD107a (*p* = 0.008), as well as Fas/CD95 (*p* = 0.01). While the number of Tregs was decreased post-vaccination (CD4^+^, CD25^high^, CD27^low^), this decrease did not reach statistical significance (Figure 1C).

### hTERT specificity of T cell response

**FIGURE 2:**
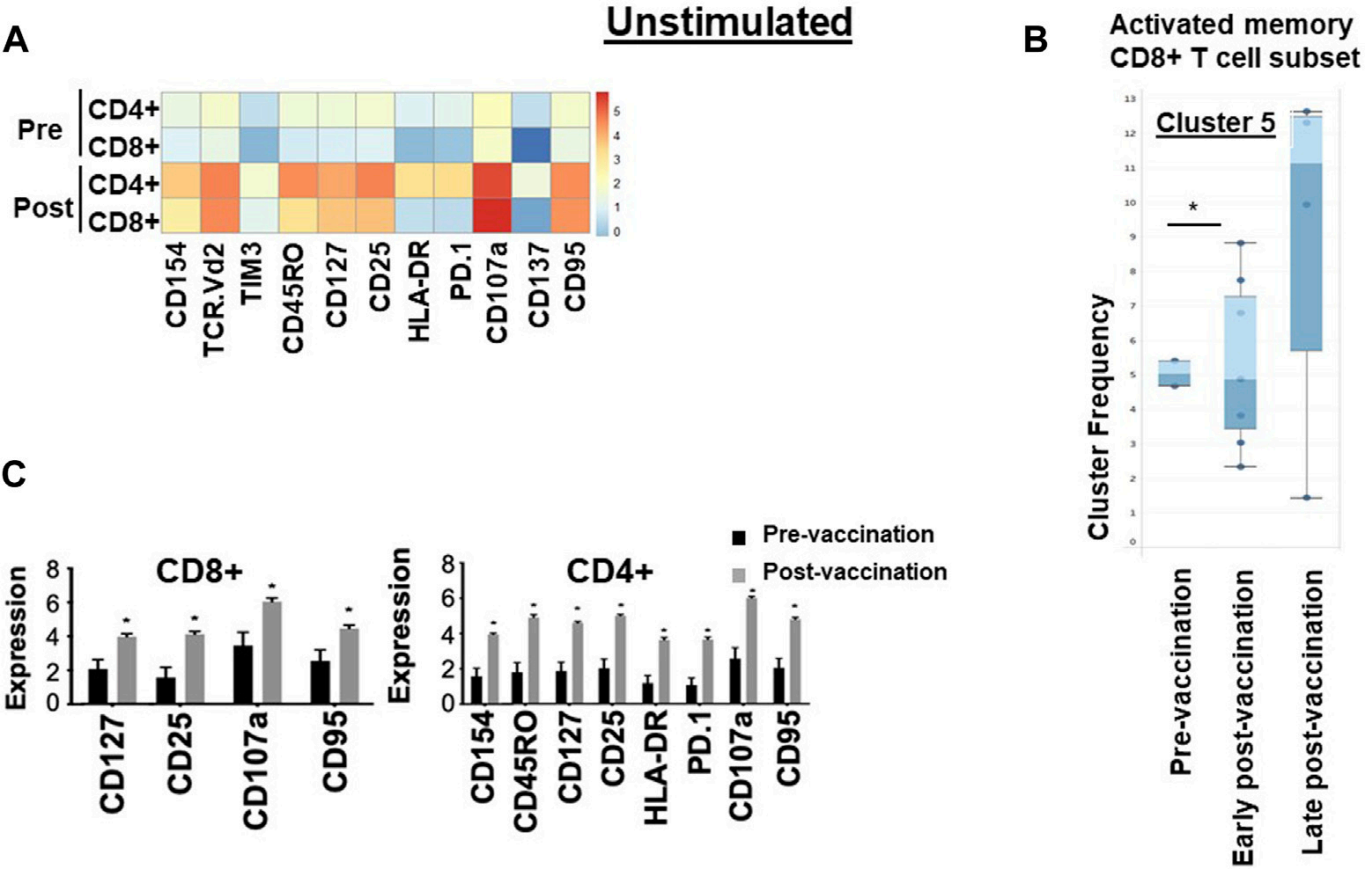
Immunological signature of PBMCs from patients in the VAPER study. **(A)** Median expression of the selected markers in pre- and post-vaccination PBMCs from 24 patients as identified by clustering algorithm CytoClustR on a subset of 400,000 cells proportionally selected from all samples. Each row represents a cell cluster and each column represents a marker. Heatmap plot generated from SPADE analysis. Expression values were transformed using the arcsin function in a cofactor of 5. The plot shows a difference in the immunological signature between pre- and post-vaccination T lymphocytes, a phenotype that shows increased presence of memory (CD45RO) activated (CD107a, HLA.DR) CD8^+^ T cells. These stimulated cells appear to express inhibitory co-receptors (PD-1, TIM3), as well as CD95 (APO-1/Fas), which are known to be increased in post-vaccination T cells. **(B,C)** Quantitation of T cell activation markers in different clusters of stimulated cells, with significantly increased expression post-vaccination in two clusters of cells (cluster 5 and cluster 7).Cluster 5: CD8^hi^, HLADR^hi^, PD-1^hi^, CTLA4^hi^, TIM-3^hi^ TCRVD2^hi^ CD45RO^hi^ CD137^hi^ CD107a^hi^, IL-2^hi^, TNF-a^hi^, Cluster 7: CD4^hi^, HLADR^hi^, PD-1^hi^, CTLA4^hi^, TIM-3^hi^ CD45RO^hi^ CD107a^hi^, IL-2^hi^Error bars are standard error of mean. Two-way Anova analysis of variance test was used for statistical analysis. *p < 0.05. The re-challenged cells showed a similar phenotype with unchallenged cells; in addition re-challenged cells also expressed significantly higher cytokines TNF-a, IFN-g and IL-2 after vaccination.

After *in vitro* overnight stimulation with hTERT, or an irrelevant peptide as control, the proportions of both CD4^+^ and CD8^+^ T cells expressing cytokines IL-2 (*p* = 0.01, *p* = 0.02), TNF-α (*p* = 0.03/*p* = 0.02) and IFN-γ (*p* = 0.03/*p* = 0.02) increased significantly in response to re-challenge, compared to pre-vaccination levels (Figure 2), but not in response to the irrelevant peptide (Supplementary Figure S3). There were no changes in NK, γδ T cell or Treg populations following vaccination (Supplementary Figure S4).

To confirm these findings and to eliminate any bias, automated unsupervised clustering PhenoGraph was applied independently and it further confirmed these observations in both unchallenged (Figure 1B) and the *in vitro* re-challenged (Figure 2C) PBMC samples. Unsupervised clustering PhenoGraph has identified a cluster of cells, the phenotype of which has altered after vaccination. Subsequent statistical analysis of these cell clusters showed that the frequency of some of these cell clusters changed after vaccination. The most notable of these changes is a significant increase in the population of activated memory CD8^+^ T cells in the unchallenged PBMCs (Figure 1B). A further overnight *in vitro* stimulation of the PBMCs with the hTERT peptides present in the VAPER vaccine identified additional populations of T cell subsets with significantly enhanced antigen-specific activation markers in both memory CD4 and CD8 T cells as presented in Figure 2. The re-challenged cells also expressed significantly higher cytokines TNF-α, IFN- γ and IL-2 after vaccination. In contrast with the phenotype of T cells exposed to hTERT peptides *ex vivo*, stimulation with the irrelevant peptide RMF did not result in CD8^+^ or CD4^+^ T cell expansion, nor was there an increase in T cell subsets displaying activation, memory or apoptotic markers in the post-vaccination samples (data not shown). This suggests that expansion after vaccination was substantially hTERT-specific.

### The antigen specificity of expanded T cells

**FIGURE 3:**
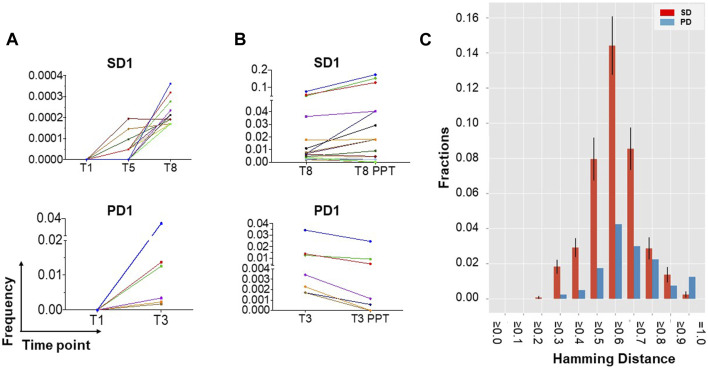
TCR-β sequencing identifies the emergence of new clonal populations of T cells after hTERT vaccination. **(A)** New clonal populations of T cells emerge after vaccination in patients with both maintained stable disease (SD) and disease progression (PD). DNA was extracted from fresh frozen PBMCs collected prior to and after vaccination and analysed for TCR-β oligoclonality. TCR sequence analysis identifies the emergence of oligoclonal populations of T cells at the frequencies indicated, the 20 most prevalent of which are presented for 1 patient each with SD or with PD, representative of a total of 6 SD and 2 PD patients analyzed. Time points are indicated in weeks from starting vaccination (for example, T8 = week 8). **(B)** Evidence for hTERT specificity among expanded T cells clones. TCR-β oligoclonality was further analysed following 2 weeks of *ex vivo* stimulation with hTERT peptides, in order to assess T cell specificity for hTERT. hTERT-driven expansion of a subset of the oligoclonal T cells is most evident in patients with maintained SD rather than PD. **(C)** Sequence similarity analysis (distance score by Hamming metric) supports true hTERT specificity of T cell clones responding to *ex vivo* culture with hTERT peptide. The Hamming distance (HD) amongst the peptide sequences encoded by the CDR3 region of TCR-β was measured in the 20 most prevalent clones of T cells that had expanded after *in vitro* stimulation with hTERT. Samples from six patients with stable disease (SD; red bars) are compared with those from two with more rapidly progressing disease (PD; blue bars). Mean and standard deviation values for HD are shown ≥ the corresponding x-axis value. Error bars are calculated only for the SD group, which includes more than one inter-patient pairwise comparison. HD is scaled between zero (identity) and one (no similarity). The left-skewness of the PD group distribution and higher fractions of lower HD values for the SD group indicates a strong convergence at the sequence level for patients with SD, and therefore a higher similarity in the MHC antigen targets recognized by the oligoclonally expanded T cells, compared to the PD group.

TCR Vβ CDR3 sequencing of patients’ T cells was performed, pre- and post-vaccination, to assess the antigen-specificity of responses to vaccination. Six patients with maintained stable disease (SD) and two with early disease progression (PD) were selected for study. TCR-β sequence analysis of PBMC DNA revealed the emergence of oligoclonal populations of T cells after hTERT vaccination in both SD and PD patients. The 20 most prevalent clones are presented in Figure 3A and Supplementary Figure S5A.

To investigate the specificity of TCR clones, TCR-β clonality was also analysed after two rounds of *ex vivo* stimulation with hTERT peptides over 2 weeks, in the absence of any adjuvants. This data showed further hTERT-driven expansion of a subset of the oligoclonal T cells which was predominantly evident in patients with maintained stable disease (median: 6.9%; range: 1.1%–23%) compared to patients with progressive disease (3.1%), providing evidence for hTERT specificity of a subset of the clonally expanded T cells (Figure 3B; Supplementary Figure S5B). We then examined TCR similarity, among the T cell clones that had increased in frequency after *in vitro* hTERT stimulation, by calculating the pairwise Hamming distances across the CDR3 sequences of these clones, as a measure of the likelihood of two separate TCRs recognising the same HLA/antigen complex. The Hamming distances amongst CDR3 sequences in the top 20 hTERT-expanded T cell clones, i.e., the most prevalent clones were substantially shorter in the vaccinated patients who maintained stable disease (SD) compared with patients who had developed a progressive disease. These results provide a strong indication of similarity between different TCR clones in the group with SD, and further support for hTERT-specificity of oligoclonally expanded T cells appearing after vaccination (Figure 3C).

## Discussion

hTERT is overexpressed in >90% of cancer cells but not in normal tissues, apart from stem cells and mitotically active normal cell populations [1–3, 21–27]; therefore representing an attractive tumour antigen target for therapeutic vaccination in a range of cancers [28]. hTERT peptides are naturally processed by tumour cells, and are presented in the context of HLA class-I and -II molecules [29]. Therapeutic hTERT peptide vaccination has been previously investigated in patients with cancer, and shown to be safe [30]. Clinical responses have been variable [31–33], but vaccination-induced stimulation of prominent specific T cell responses have been previously documented [9, 34–36], and hTERT peptides bind with high affinity to particular HLA haplotypes. However, class-I binding hTERT peptides used in previous trials have limited therapeutic applicability because of HLA haplotype heterogeneity in patients [4, 33, 37–39]. In this trial we have used a combination of peptides selected for high binding affinities to a number of commonly expressed HLA haplotypes. This strategy aimed to ensure that at least one component of the peptide library is likely to be effectively presented by the HLAs that are present in 90% of European patients. HLA haplotyping was not necessary to use this treatment; we selected a pool of seven hTERT peptides to ensure presentation by both HLA class-I and class-II in 90% of patients. For the future studies, a wider mixture of peptides that can be presented by different HLA classes, combined with suitable adjuvants, can possibly be used to cover a larger population of patients.

We applied the TLR-7 agonist imiquimod topically, with the aim of promoting antigen uptake by dendritic cells (DCs) and activation of the release of pro-inflammatory cytokines [40]. Stimulation of immature DCs with TLR agonists upregulates CCR7, increasing their migration to draining lymph nodes [41]. Animal models have shown that imiquimod can enhance dendritic cell survival, as well as promoting tumour–specific T cell priming, trafficking and accumulation in lymph nodes [42, 43]. Appropriately formulated adjuvants such as Montanide provide a depot for the prolonged release of antigens, preventing rapid degradation, and ensuring a continuous delivery of antigen to the regional lymph nodes. Trials using Montanide have demonstrated significant enhancement of T cell immune responses and improved clinical outcomes [44, 45]. Combination of hTERT peptides with Montanide has previously led to the induction of effective CD8^+^ T cell responses [46]. Metronomic low dose cyclophosphamide depletes Tregs whilst preserving overall lymphocyte numbers [47], and augmented effector T cell responses are also reported [48–51].

The vaccination strategy developed here is safe, with hypersensitivity to the Montanide/peptide mix being the only adverse event warranting treatment discontinuation. There were no RECIST responses in this phase 1 trial conducted in patients with therapy-resistant, metastatic disease. Despite their advanced cancers, maintained stable disease for at least 6 months was observed in 24% of patients, with a range of tumour types (colorectal, lung, pancreas, prostate, breast, cervix, ovary, upper GI and pleural).

Strong CD4^+^ and CD8^+^ T cell responses were evident following vaccination with this hTERT peptide mixture. Using an unbiased clustering method, we show that these expanded T cells have an activated phenotype but that they also express markers of exhaustion and apoptosis. This data suggests that combining hTERT vaccination with immune checkpoint blockade may further improve the magnitude and longevity of the overall immune response. Interestingly, we did not find a significant change in the frequency or phenotype of other immune cell populations such as NK cells, γδ T cells or Tregs after vaccination. A numerical reduction in Tregs did not reach statistical significance, possibly due to small sample size.

The expanded T cells showed far less sequence diversity in their CDR3 region as evident by the number of expanded clones post-vaccination. The dominant clones were further expanded following *in vitro* rechallenge with hTERT peptides but not the irrelevant peptide, indicating the hTERT-specificity of the clonally expanded T cells. However, considering the variety of HLA haplotype in these patients, finding similar CDR3 sequences to confirm that T cells from different patients share specificity is challenging. To overcome this issue, we calculated the Hamming-distance similarity index For the largest expanded clones as a proxy for convergence. Sequence convergence indicates sequence similarity between the CDR3 regions of different oligoclonally expanded T cell populations, supporting the conclusion that the expanded T cell clones from different patients have been selected to detect the same HLA-peptide complexes.

This trial is a first step in the clinical development of a cancer vaccine using a strategy that has been termed “Combined Adjuvants for Synergistic Activation of Cellular immunity” (CASAC) [18, 19]. Our pharmacodynamic data indicates that this vaccination strategy induces immunological responses against a tumour-associated self-antigen, hTERT. Specifically, we have demonstrated that hTERT peptide vaccination, in combination with adjuvants and metronomic cyclophosphamide therapy, can generate activated hTERT-specific CD4+/CD8+ T effector responses against this tumour associated antigen in cancer patients with therapy-resistant solid tumours. However, the best clinical responses seen in this trial were prolonged stable disease, and no tumour shrinkage by RECIST criteria occurred, possibly because of persistent PD1-positive Tregs. Future combination of this vaccination strategy with PD-1/PD-L1-targeted immune checkpoint inhibition may improve clinical efficacy, as has been observed in other pairings of vaccination with checkpoint inhibitors [52–56].

## Conclusion

We conclude that this vaccine combination is associated with antigen-specific immunological responses and can generate activated hTERT-specific CD4+/CD8+ effector responses against this tumour associated antigen in patients with therapy-resistant solid tumours. Clinical response could be improved by combination with anti-PD1 checkpoint inhibition to address the emergence of an exhausted T cell population.

## Data Availability

The datasets presented in this study can be found in online repositories. The names of the repository/repositories and accession number(s) can be found in the article/Supplementary Material.

## Footnotes

1
http://www.syfpeithi.de/

2 Available at: https://github.com/kordastilab/cytoClustR

## References

[1] Kim NW. Clinical implications of telomerase in cancer. Eur J Cancer (1997) 33(5):781–6. 10.1016/s0959-8049(97)00057-9 9282117

[2] Kim NW Piatyszek MA Prowse KR Harley CB West MD Ho PLC Specific association of human telomerase activity with immortal cells and cancer. Science (1994) 266(5193):2011–5. 10.1126/science.7605428 7605428

[3] Shay JW Wright WE. The reactivation of telomerase activity in cancer progression. Trends Genet (1996) 12(4):129–31. 10.1016/0168-9525(96)30018-8 8901415

[4] Lilleby W Gaudernack G Brunsvig PF Vlatkovic L Schulz M Mills K Phase I/IIa clinical trial of a novel hTERT peptide vaccine in men with metastatic hormone-naive prostate cancer. Cancer Immunol Immunother (2017) 66(7):891–901. 10.1007/s00262-017-1994-y 28391357 PMC11028648

[5] Mizukoshi E Nakagawa H Kitahara M Yamashita T Arai K Sunagozaka H Immunological features of T cells induced by human telomerase reverse transcriptase-derived peptides in patients with hepatocellular carcinoma. Cancer Lett (2015) 364(2):98–105. 10.1016/j.canlet.2015.04.031 25982205

[6] Mehrotra S Britten CD Chin S Garrett-Mayer E Cloud CA Li M Vaccination with poly(IC:LC) and peptide-pulsed autologous dendritic cells in patients with pancreatic cancer. J Hematol Oncol (2017) 10(1):82. 10.1186/s13045-017-0459-2 28388966 PMC5384142

[7] Bijker MS van den Eeden SJF Franken KL Melief CJM Offringa R van der Burg SH. CD8+ CTL priming by exact peptide epitopes in incomplete Freund's adjuvant induces a vanishing CTL response, whereas long peptides induce sustained CTL reactivity. J Immunol (2007) 179(8):5033–40. 10.4049/jimmunol.179.8.5033 17911588

[8] Schroers R Huang XF Hammer J Zhang J Chen SY. Identification of HLA DR7-restricted epitopes from human telomerase reverse transcriptase recognized by CD4+ T-helper cells. Cancer Res (2002) 62(9):2600–5. 11980655

[9] Minev B Hipp J Firat H Schmidt JD Langlade-Demoyen P Zanetti M. Cytotoxic T cell immunity against telomerase reverse transcriptase in humans. Proc Natl Acad Sci U S A (2000) 97(9):4796–801. 10.1073/pnas.070560797 10759561 PMC18312

[10] Vonderheide RH Anderson KS Hahn WC Butler MO Schultze JL Nadler LM. Characterization of HLA-A3-restricted cytotoxic T lymphocytes reactive against the widely expressed tumor antigen telomerase. Clin Cancer Res (2001) 7(11):3343–8. 11705846

[11] Cortez-Gonzalez X Sidney J Adotevi O Sette A Millard F Lemonnier F Immunogenic HLA-B7-restricted peptides of hTRT. Int Immunol (2006) 18(12):1707–18. 10.1093/intimm/dxl105 17077179

[12] Bernardeau K Kerzhero J Fortun A Moreau-Aubry A Favry E Echasserieau K A simple competitive assay to determine peptide affinity for HLA class II molecules: a useful tool for epitope prediction. J Immunological Methods (2011) 371(1-2):97–105. 10.1016/j.jim.2011.06.018 21729700

[13] Goldinger SM Dummer R Baumgaertner P Mihic‐Probst D Schwarz K Hammann‐Haenni A Nano-particle vaccination combined with TLR-7 and -9 ligands triggers memory and effector CD8(+) T-cell responses in melanoma patients. Eur J Immunol (2012) 42(11):3049–61. 10.1002/eji.201142361 22806397 PMC3549564

[14] Veltman JD Lambers MEH van Nimwegen M de Jong S Hendriks RW Hoogsteden HC Low-dose cyclophosphamide synergizes with dendritic cell-based immunotherapy in antitumor activity. J Biomed Biotechnol (2010) 2010:1–10. 10.1155/2010/798467 PMC2874992 20508851

[15] Greten TF Ormandy LA Fikuart A Höchst B Henschen S Hörning M Low-dose cyclophosphamide treatment impairs regulatory T cells and unmasks AFP-specific CD4+ T-cell responses in patients with advanced HCC. J Immunother (2010) 33(2):211–8. 10.1097/cji.0b013e3181bb499f 20139774

[16] Fontana A Bocci G Galli L D'Arcangelo M Derosa L Fioravanti A Metronomic cyclophosphamide in elderly patients with advanced, castration-resistant prostate cancer. J Am Geriatr Soc (2010) 58(5):986–8. 10.1111/j.1532-5415.2010.02833.x 20722827

[17] Sevko A Sade-Feldman M Kanterman J Michels T Falk CS Umansky L Cyclophosphamide promotes chronic inflammation-dependent immunosuppression and prevents antitumor response in melanoma. J Invest Dermatol (2013) 133(6):1610–9. 10.1038/jid.2012.444 23223128

[18] Tye GJ Ioannou K Amofah E Quartey-Papafio R Westrop SJ Krishnamurthy P The combined molecular adjuvant CASAC enhances the CD8+ T cell response to a tumor-associated self-antigen in aged, immunosenescent mice. Immun Ageing (2015) 12:6. 10.1186/s12979-015-0033-0 26157468 PMC4495856

[19] Wells JW Cowled CJ Farzaneh F Noble A. Combined triggering of dendritic cell receptors results in synergistic activation and potent cytotoxic immunity. J Immunol (2008) 181(5):3422–31. 10.4049/jimmunol.181.5.3422 18714014

[20] MacKay DJC. Information theory, inference, and learning algorithms. Cambridge, UK; New York: Cambridge University Press xii (2003). p. 628.

[21] Nakamura TM Morin GB Chapman KB Weinrich SL Andrews WH Lingner J Telomerase catalytic subunit homologs from fission yeast and human. Science (1997) 277(5328):955–9. 10.1126/science.277.5328.955 9252327

[22] Shay JW Gazdar AF. Telomerase in the early detection of cancer. J Clin Pathol (1997) 50(2):106–9. 10.1136/jcp.50.2.106 9155689 PMC499733

[23] Umbricht CB Sherman ME Dome J Carey LA Marks J Kim N Telomerase activity in ductal carcinoma *in situ* and invasive breast cancer. Oncogene (1999) 18(22):3407–14. 10.1038/sj.onc.1202714 10362362

[24] Ahmed A Tollefsbol TO. Telomerase, telomerase inhibition, and cancer. J Anti-Aging Med (2003) 6(4):315–25. 10.1089/109454503323028911 15142433

[25] Castelo-Branco P Choufani S Mack S Gallagher D Zhang C Lipman T Methylation of the TERT promoter and risk stratification of childhood brain tumours: an integrative genomic and molecular study. Lancet Oncol (2013) 14(6):534–42. 10.1016/s1470-2045(13)70110-4 23598174

[26] Koziel JE Fox MJ Steding CE Sprouse AA Herbert BS. Medical genetics and epigenetics of telomerase. J Cell Mol Med (2011) 15(3):457–67. 10.1111/j.1582-4934.2011.01276.x 21323862 PMC3922369

[27] Ramakrishnan S Eppenberger U Mueller H Shinkai Y Narayanan R. Expression profile of the putative catalytic subunit of the telomerase gene. Cancer Res (1998) 58(4):622–5. 9485011

[28] Vonderheide RH. Prospects and challenges of building a cancer vaccine targeting telomerase. Biochimie (2008) 90(1):173–80. 10.1016/j.biochi.2007.07.005 17716803 PMC2745192

[29] Inderberg-Suso EM Trachsel S Lislerud K Rasmussen AM Gaudernack G. Widespread CD4+ T-cell reactivity to novel hTERT epitopes following vaccination of cancer patients with a single hTERT peptide GV1001. Oncoimmunology (2012) 1(5):670–86. 10.4161/onci.20426 22934259 PMC3429571

[30] Brunsvig PF Aamdal S Gjertsen MK Kvalheim G Markowski-Grimsrud CJ Sve I Telomerase peptide vaccination: a phase I/II study in patients with non-small cell lung cancer. Cancer Immunol Immunother (2006) 55(12):1553–64. 10.1007/s00262-006-0145-7 16491401 PMC11030882

[31] Bernhardt SL Gjertsen MK Trachsel S Møller M Eriksen JA Meo M Telomerase peptide vaccination of patients with non-resectable pancreatic cancer: a dose escalating phase I/II study. Br J Cancer (2006) 95(11):1474–82. 10.1038/sj.bjc.6603437 17060934 PMC2360729

[32] Vetsika EK Konsolakis G Aggouraki D Kotsakis A Papadimitraki E Christou S Immunological responses in cancer patients after vaccination with the therapeutic telomerase-specific vaccine Vx-001. Cancer Immunol Immunother (2012) 61(2):157–68. 10.1007/s00262-011-1093-4 21858533 PMC11028568

[33] Bolonaki I Kotsakis A Papadimitraki E Aggouraki D Konsolakis G Vagia A Vaccination of patients with advanced non-small-cell lung cancer with an optimized cryptic human telomerase reverse transcriptase peptide. J Clin Oncol (2007) 25(19):2727–34. 10.1200/jco.2006.10.3465 17602077

[34] Arai J Yasukawa M Ohminami H Kakimoto M Hasegawa A Fujita S. Identification of human telomerase reverse transcriptase-derived peptides that induce HLA-A24-restricted antileukemia cytotoxic T lymphocytes. Blood (2001) 97(9):2903–7. 10.1182/blood.v97.9.2903 11313288

[35] Vonderheide RH Hahn WC Schultze JL Nadler LM. The telomerase catalytic subunit is a widely expressed tumor-associated antigen recognized by cytotoxic T lymphocytes. Immunity (1999) 10(6):673–9. 10.1016/s1074-7613(00)80066-7 10403642

[36] Aloysius MM Mc Kechnie AJ Robins RA Verma C Eremin JM Farzaneh F Generation *in vivo* of peptide-specific cytotoxic T cells and presence of regulatory T cells during vaccination with hTERT (class I and II) peptide-pulsed DCs. J Transl Med (2009) 7:18. 10.1186/1479-5876-7-18 19298672 PMC2674878

[37] Greten TF Forner A Korangy F N'Kontchou G Barget N Ayuso C A phase II open label trial evaluating safety and efficacy of a telomerase peptide vaccination in patients with advanced hepatocellular carcinoma. BMC Cancer (2010) 10:209. 10.1186/1471-2407-10-209 20478057 PMC2882353

[38] Kotsakis A Papadimitraki E Vetsika EK Aggouraki D Dermitzaki EK Hatzidaki D A phase II trial evaluating the clinical and immunologic response of HLA-A2(+) non-small cell lung cancer patients vaccinated with an hTERT cryptic peptide. Lung Cancer (2014) 86(1):59–66. 10.1016/j.lungcan.2014.07.018 25130084

[39] Menez-Jamet J Gallou C Rougeot A Kosmatopoulos K. Optimized tumor cryptic peptides: the basis for universal neo-antigen-like tumor vaccines. Ann Transl Med (2016) 4(14):266. 10.21037/atm.2016.05.15 27563653 PMC4971378

[40] Shi M Chen X Ye K Yao Y Li Y. Application potential of toll-like receptors in cancer immunotherapy: systematic review. Medicine (Baltimore) (2016) 95(25):e3951. 10.1097/md.0000000000003951 PMC4998329 27336891

[41] Gunn MD Kyuwa S Tam C Kakiuchi T Matsuzawa A Williams LT Mice lacking expression of secondary lymphoid organ chemokine have defects in lymphocyte homing and dendritic cell localization. J Exp Med (1999) 189(3):451–60. 10.1084/jem.189.3.451 9927507 PMC2192914

[42] Rechtsteiner G Warger T Osterloh P Schild H Radsak MP. Cutting edge: priming of CTL by transcutaneous peptide immunization with imiquimod. J Immunol (2005) 174(5):2476–80. 10.4049/jimmunol.174.5.2476 15728450

[43] Prins RM Craft N Bruhn KW Khan-Farooqi H Koya RC Stripecke R The TLR-7 agonist, imiquimod, enhances dendritic cell survival and promotes tumor antigen-specific T cell priming: relation to central nervous system antitumor immunity. J Immunol (2006) 176(1):157–64. 10.4049/jimmunol.176.1.157 16365406

[44] Aucouturier J Ascarateil S Dupuis L. The use of oil adjuvants in therapeutic vaccines. Vaccine (2006) 24(S2):44–5. 10.1016/j.vaccine.2005.01.116 16823921

[45] Karbach J Gnjatic S Bender A Neumann A Weidmann E Yuan J Tumor‐reactive CD8^+^ T‐cell responses after vaccination with NY‐ESO‐1 peptide, CpG 7909 and Montanide® ISA‐51: association with survival. Int J Cancer (2010) 126(4):909–18. 10.1002/ijc.24850 19728336

[46] Mavroudis D Bolonakis I Cornet S Myllaki G Kanellou P Kotsakis A A phase I study of the optimized cryptic peptide TERT(572y) in patients with advanced malignancies. Oncology (2006) 70(4):306–14. 10.1159/000096252 17047402

[47] Ghiringhelli F Menard C Puig PE Ladoire S Roux S Martin F Metronomic cyclophosphamide regimen selectively depletes CD4+CD25+ regulatory T cells and restores T and NK effector functions in end stage cancer patients. Cancer Immunol Immunother (2007) 56(5):641–8. 10.1007/s00262-006-0225-8 16960692 PMC11030569

[48] Radojcic V Bezak KB Skarica M Pletneva MA Yoshimura K Schulick RD Cyclophosphamide resets dendritic cell homeostasis and enhances antitumor immunity through effects that extend beyond regulatory T cell elimination. Cancer Immunol Immunother (2010) 59(1):137–48. 10.1007/s00262-009-0734-3 19590872 PMC3103867

[49] Li Y Lu W Yang J Edwards M Jiang S. Survivin as a biological biomarker for diagnosis and therapy. Expert Opin Biol Ther (2021) 21(11):1429–41. 10.1080/14712598.2021.1918672 33877952

[50] Negrini S De Palma R Filaci G. Anti-cancer immunotherapies targeting telomerase. Cancers (Basel) (2020) 12(8):2260. 10.3390/cancers12082260 32806719 PMC7465444

[51] Fenoglio D Traverso P Parodi A Tomasello L Negrini S Kalli F A multi-peptide, dual-adjuvant telomerase vaccine (GX301) is highly immunogenic in patients with prostate and renal cancer. Cancer Immunol Immunother (2013) 62(6):1041–52. 10.1007/s00262-013-1415-9 23591981 PMC11029691

[52] Curran MA Glisson BS. New hope for therapeutic cancer vaccines in the era of immune checkpoint modulation. Annu Rev Med (2019) 70:409–24. 10.1146/annurev-med-050217-121900 30379596

[53] Chung V Kos FJ Hardwick N Yuan Y Chao J Li D Evaluation of safety and efficacy of p53MVA vaccine combined with pembrolizumab in patients with advanced solid cancers. Clin Transl Oncol (2019) 21(3):363–72. 10.1007/s12094-018-1932-2 30094792 PMC8802616

[54] Massarelli E William W Johnson F Kies M Ferrarotto R Guo M Combining immune checkpoint blockade and tumor-specific vaccine for patients with incurable human papillomavirus 16-related cancer: a phase 2 clinical trial. JAMA Oncol (2019) 5(1):67–73. 10.1001/jamaoncol.2018.4051 30267032 PMC6439768

[55] Weber JS Kudchadkar RR Yu B Gallenstein D Horak CE Inzunza HD Safety, efficacy, and biomarkers of nivolumab with vaccine in ipilimumab-refractory or -naive melanoma. J Clin Oncol (2013) 31(34):4311–8. 10.1200/jco.2013.51.4802 24145345 PMC3837092

[56] Gibney GT Kudchadkar RR DeConti RC Thebeau MS Czupryn MP Tetteh L Safety, correlative markers, and clinical results of adjuvant nivolumab in combination with vaccine in resected high-risk metastatic melanoma. Clin Cancer Res (2015) 21(4):712–20. 10.1158/1078-0432.ccr-14-2468 25524312 PMC4620684

