# Supplementary figures and images for: A phase 1 trial of human telomerase reverse transcriptase (hTERT) vaccination combined with therapeutic strategies to control immune-suppressor mechanisms
Source: Exp Biol Med (Maywood). 2024 Jan 31;249:10021. doi: 10.3389/ebm.2024.10021 (PMC10911124; doi:10.3389/ebm.2024.10021)

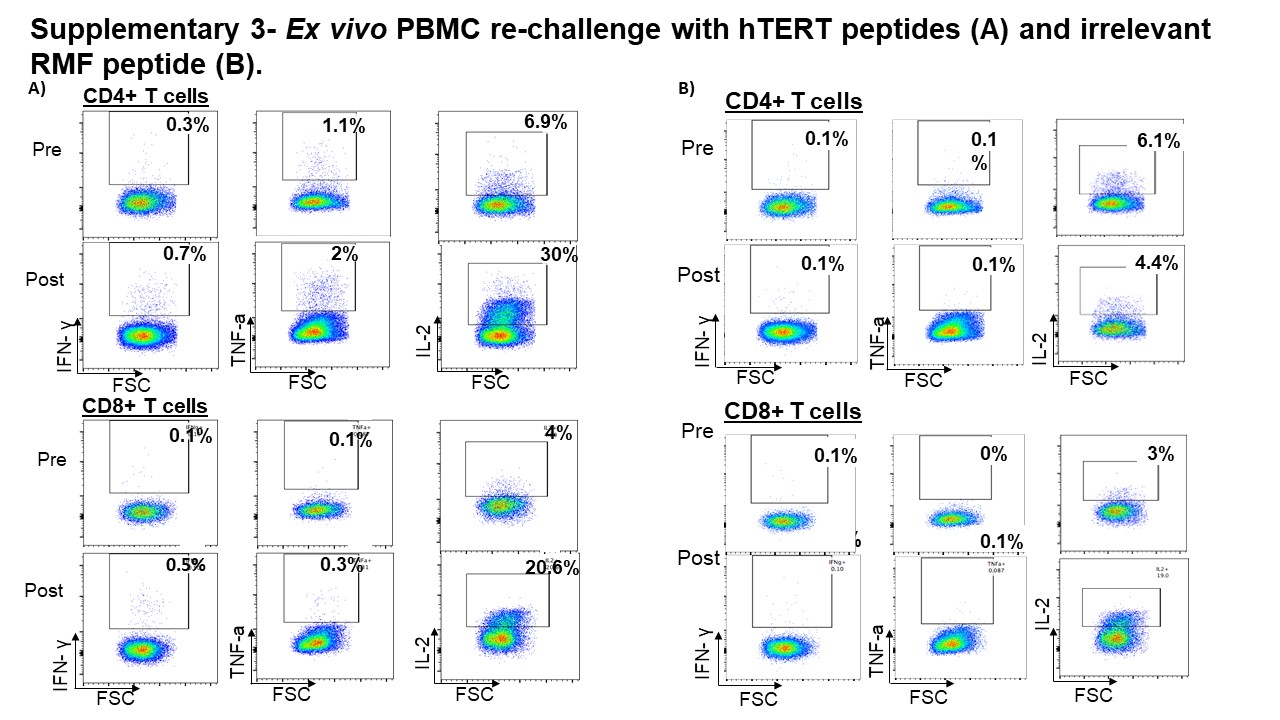

Supplement: Supplementary file 1 [file Image3.jpeg]

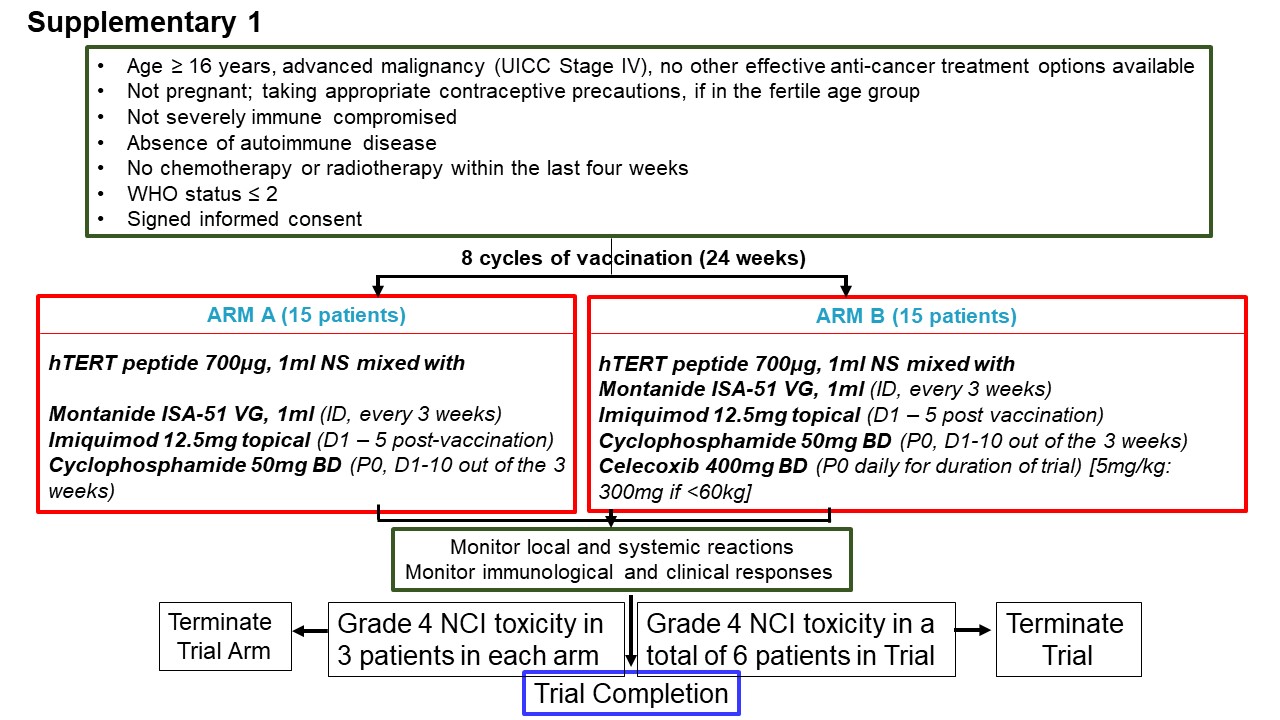

Supplement: Supplementary file 2 [file Image1.jpeg]

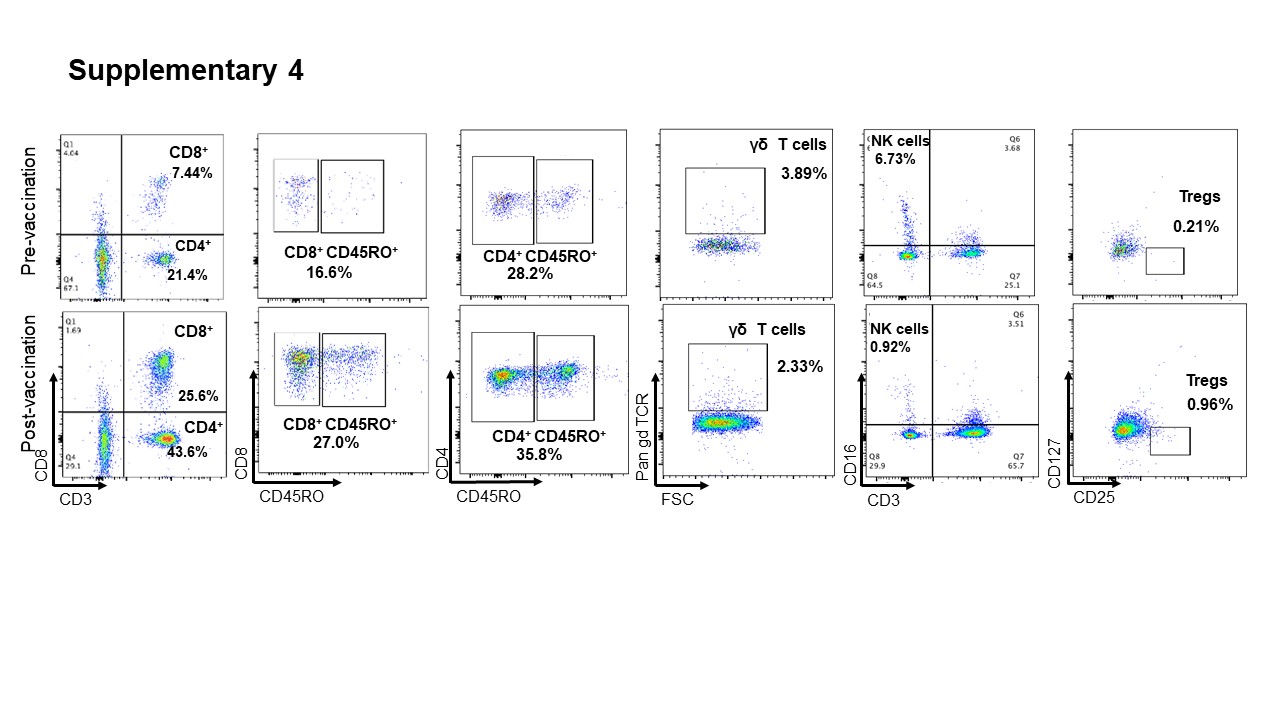

Supplement: Supplementary file 3 [file Image4.jpeg]

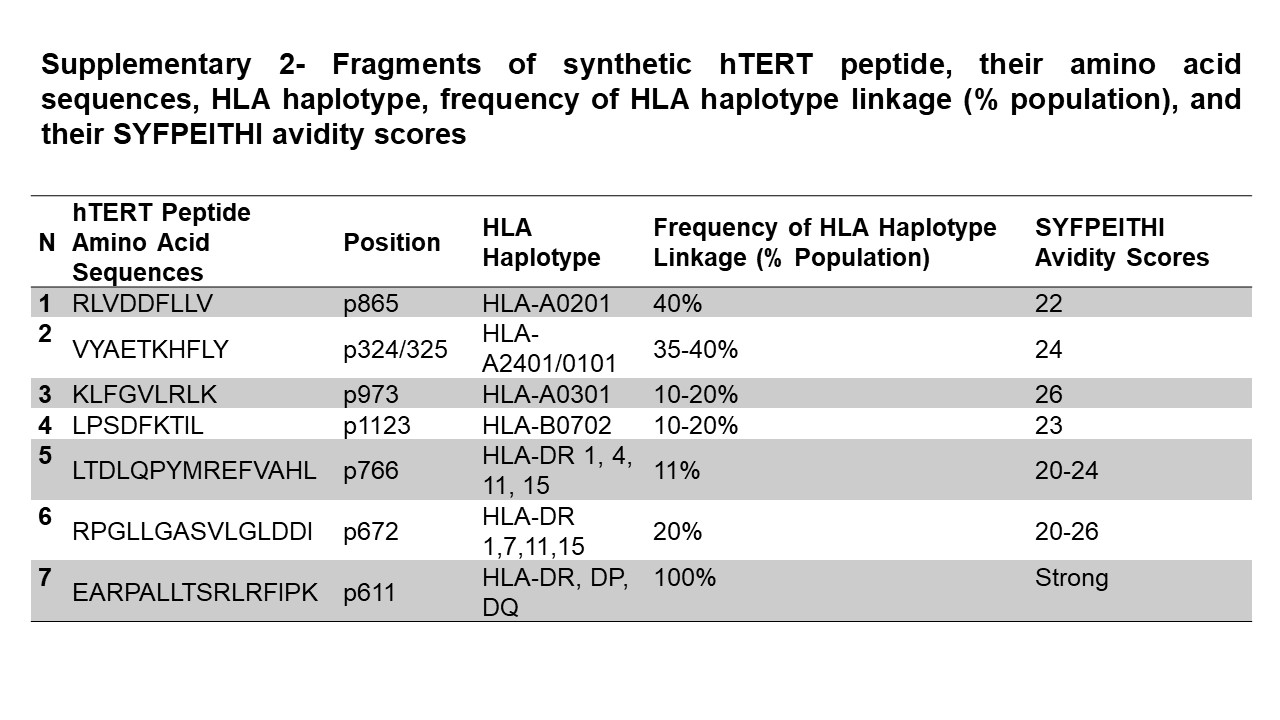

Supplement: Supplementary file 4 [file Image2.jpeg]

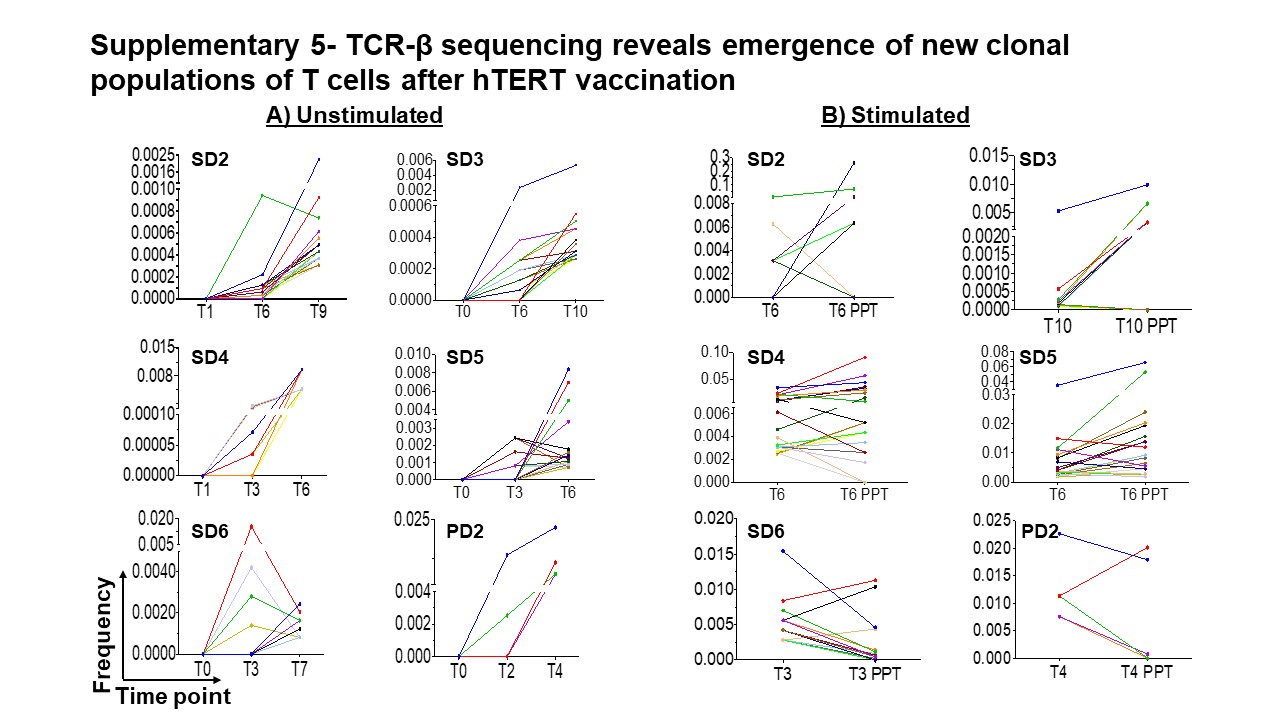

Supplement: Supplementary file 5 [file Image5.jpeg]
