# Supplementary material for: A phase 1 trial of human telomerase reverse transcriptase (hTERT) vaccination combined with therapeutic strategies to control immune-suppressor mechanisms
Source: Exp Biol Med (Maywood). 2024 Jan 31;249:10021. doi: 10.3389/ebm.2024.10021 (PMC10911124; doi:10.3389/ebm.2024.10021)
Supplement: Supplementary file 6 [file DataSheet1.pdf]

Supplementary 12- Materials and Methods

Immunophenotyping

Peripheral blood mononuclear cells (PBMCs) were isolated by the Ficoll-Paque method from peripheral blood of patients and cryopreserved in FBS and 10% DMSO prior to *ex vivo* use. The phenotypic T lymphocyte profiles were analysed *ex vivo*, pre and post vaccination, to identify activated T effector cells and checkpoint inhibitors (CIs). Cells were fixed, permeabilized and stained using a panel of antibodies against surface and intracellular markers to identify different subsets of T cells, cytokines and other relevant markers (**Supplementary 4**).

Antibodies were purchased from either BD or Biolegend, UK. Data were acquired by BD FACSymphony (Beckman Dickson, US) and analysed by a multi-parameter phenotyping strategy using the Cytobank web-based software.

Data processing, scale transformation, automated clustering, and distance computations

Data were initially processed and analyzed by Cytobank [1] using a combination of automated dimension reduction and clustering methods, including *t*-distributed stochastic neighbour embedding (tSNE) to visually (viSNE) identify the delineated subpopulations [2] as well as downstream clustering including spanning-tree progression analysis of density-normalized events (SPADE) [3] and PhenoGraph [4]. Proportional cell numbers were sampled for unsupervised viSNE analysis. The deep immunophenotyping of T cell clusters was performed using our in-house designed pipeline, the automated clustering algorithm (publicly available at: https://github.com/kordastilab/cytoClustR). Phenograph was run 30

times for parameter K (number of nearest neighbors) set from 5 to 150 with a step of 5. With increasing k, phenograph converges to about 19 clusters for the stimulated panel (at k = 75) and 16 clusters for the unstimulated panel (at k = 150). k = 100 was selected for the percentage tables and individual heatmaps (for each file). Expression values were transformed using the arcsinh function using a cofactor of 5.

TCR sequencing

The potential antigen specificity of the T cell response was evaluated by TCR-β sequencing and hamming-distance-based similarity index pre-vaccination (time point T1) and post-vaccination time points (range: T2-T10, median: T6) in patients with progressive disease (PD) and in patients in which disease progression had stabilised after vaccination (SD). Amplification and sequencing of TCR-β complementarity determining region 3 (CDR3) was performed prior to and post vaccination on genomic DNA. DNA was extracted (Qiagen's DNeasy mini-columns, QIAGEN Inc.) from whole PBMC (unstimulated) and after 2 rounds of stimulation with hTERT peptides over 2-weeks (stimulated) (see *ex vivo* T-cell stimulation assay methodology). All TCR-β characterization was performed by Adaptive Biotechnologies Corp (Seattle, WA) using the ImmunoSEQ TCR-β human assay. Samples were quantified using Dropsense96 and diluted for library preparation to standard concentration. The somatically rearranged CDR3 regions were amplified from genomic DNA using a two-step, amplification bias-controlled multiplex PCR approach [5, 6]. The first PCR consists of forward and reverse amplification primers specific for every V and J gene segment, and amplifies the hypervariable CDR3 of the immune receptor locus. The second PCR added a proprietary barcode sequence and Illumina® adapter sequences [7]. CDR3 libraries were sequenced on an Illumina instrument according to the manufacturer's

instructions. Raw sequence reads were demultiplexed according to Adaptive's proprietary barcode sequences. Demultiplexed reads were then further processed to remove adapter and primer sequences, identify and correct for technical errors introduced through PCR and sequencing, and remove primer dimer, germline and other contaminating sequences. The data was then filtered and clustered using both the relative frequency ratio between similar clones and a modified nearest-neighbor algorithm, to merge closely related sequences. The resulting sequences were sufficient to allow annotation of the V(N)D(N)J genes constituting each unique CDR3 and the translation of the encoded CDR3 amino acid sequence. V, D and J gene definitions were based on annotation in accordance with the IMGT database (www.imgt.org). The set of observed biological CDR3 sequences were normalized to correct for residual multiplex PCR amplification bias and quantified against a set of synthetic CDR3 sequence analogues [5].

For similarity index analysis, the Hamming distance, which indicates the similarity in CDR3 region of TCR-β, was measured. The Hamming distance was measured in the hTERT responsive clones in each of the SD and PD patients in order to measure the likely similarity in the MHC/antigen complexes that were recognised by the oligo-clonal T cell populations that were present after vaccination. Briefly, to capture a global convergence of SD repertoires, and absence of convergence (hypothetically) in PDs, we have done a non-exhaustive pairwise comparison of sequences among members of each group. Convergence indicates sequence similarity between the CDR3 regions of different oligoclonally expanded T cell populations. Therefore, before performing the hamming distance comparison among CDR3 regions, we first filtered the data and chose pairs with the same length with no insertions/ deletions, and hence a non-exhaustive pairwise comparison. Each pairwise combination met the criteria of a match, as defined by two TCR-β sequences with their inferred CDR3 region being of the same length with no gaps. The average and the standard

deviation values for Hamming distances are calculated to generate the bar chart with standard error bars.

Supplementary Figure Legends

Supplementary 1- Trial Flow Chart

Supplementary 2- Synthetic hTERT peptide fragments. The table shows the amino acid sequence, HLA haplotype, frequency of HLA haplotype linkage (% population), and their SYFPEITHI avidity scores.

Supplementary 3-*Ex vivo* PBMC re-challenge with hTERT peptides (A) and irrelevant RMF peptide (B). Flowcytometry analysis of patient PBMCs using IFN-g, TNF-a and IL2 markers.

Supplementary 4- Flowcytometry analysis of patient PBMCs and identification of memory and naïve T cells.

Supplementary 5- TCR-β sequencing reveals emergence of new clonal populations of T-cells after hTERT vaccination. A) To answer whether VAPER vaccination induces hTERT-specific T-cell responses, DNA was extracted from fresh frozen PBMCs, and analysed for TCR-β oligoclonality, in order to enumerate the frequency/prevalence of the oligoclonal populations of T-cells, prior to and after VAPER vaccination. TCR sequence analysis identifies the emergence of oligoclonal populations of T-cells, the tope 20 of which are presented in Panel A for 6 patients with stable (SD) and 2 patients with progressive disease (PD). The X axes shows the time points at which the PBMC were isolated from the patients, and the Y axes show the frequency of each clone. TCR-β sequencing identifies the emergence of new clonal populations of T-cells after hTERT vaccination in both PD and SD groups. **B)**

TCR-β oligoclonality was further analysed after 2 weeks of *in vitro* stimulation with hTERT peptides present in the VAPER vaccine, in order to assess their specificity against hTERT. The subsequent analysis of T-cell oligoclonality after 2 weekly rounds of *in vitro* stimulation with hTERT peptides (in the absence of any adjuvants), shows further hTERT driven expansion of a subset of the oligoclonal T-cells. This is predominantly evident in patients with stable disease. Such hTERT driven *in vitro* expansion of the oligoclonal T-cells is much less evident in patients with progressive disease compared to non-progressors, providing compelling evidence of hTERT specificity of a subset of the clonally expanded T-cells.

1. Kotecha, N., P.O. Krutzik, and J.M. Irish, *Web-based analysis and publication of flow cytometry experiments.* Curr Protoc Cytom, 2010. **Chapter 10**: p. Unit10 17.
2. Amir el, A.D., et al., *viSNE enables visualization of high dimensional single-cell data and reveals phenotypic heterogeneity of leukemia.* Nat Biotechnol, 2013. **31**(6): p. 545-52.
3. Qiu, P., et al., *Extracting a cellular hierarchy from high-dimensional cytometry data with SPADE.* Nat Biotechnol, 2011. **29**(10): p. 886-91.
4. Levine, J.H., et al., *Data-Driven Phenotypic Dissection of AML Reveals Progenitor-like Cells that Correlate with Prognosis.* Cell, 2015. **162**(1): p. 184-97.
5. Carlson, C.S., et al., *Using synthetic templates to design an unbiased multiplex PCR assay.* Nat Commun, 2013. **4**: p. 2680.
6. Robins, H.S., et al., *Comprehensive assessment of T-cell receptor beta-chain diversity in alphabeta T cells.* Blood, 2009. **114**(19): p. 4099-107.
7. Robins, H., et al., *Ultra-sensitive detection of rare T cell clones.* J Immunol Methods, 2012. **375**(1-2): p. 14-9.
